# SLC25A39 facilitates Sorafenib resistance in hepatocellular carcinoma by inhibiting mitochondrial oxidative stress-induced ferroptosis

**DOI:** 10.1186/s12935-025-04151-9

**Published:** 2026-01-06

**Authors:** Xilin Geng, Jibin Li, Bing Wu, Weifang Wang, Zeyu Li, Sinan Liu, Hui Li, Hulin Chang

**Affiliations:** 1 Department of Hepatobiliary Surgery, Shaanxi Provincial People’s Hospital, 256 West Friendship Road, Xi’an, 710068 China; 2 State Key Laboratory of Holistic Integrative Management of Gastrointestinal Cancers, Department of Physiology and Pathophysiology, Air Force Medical University, Xi’an, 710032 China; 3 Department of Geriatrics, the 940th Hospital of Joint Logistics Support force of Chinese People’s Liberation Army, Lanzhou, 730050 China; 4 Department of General Surgery, Shaanxi Provincial People’s Hospital, Xi’an, 710068 China; 5 Department of SICU, The First Affiliated Hospital of Xi’an Jiaotong University, Xi’an, 710061 China

**Keywords:** SLC25A39, Growth, Mitochondrial ROS, Ferroptosis, HCC

## Abstract

Mitochondria are intracellular double-membrane-bound organelles that play a crucial role in maintaining redox homeostasis, disruptions of which are closely linked to oncogenesis. Recent investigations have identified SLC25A39 as a mitochondrial membrane transporter essential for the importation of glutathione (GSH) into mitochondria. However, the roles of SLC25A39 in human malignancies remain inadequately understood. Here, we observed a marked upregulation of SLC25A39, which correlates with poor prognostic outcomes in hepatocellular carcinoma (HCC). The silencing of SLC25A39 resulted in a reduction of HCC cell viability and proliferation through inducing ferroptosis via increasing mitochondrial ROS accumulation. In contrast, the overexpression of SLC25A39 enhanced survival and proliferation of HCC cells by promoting ferroptosis evasion through reducing mitochondrial ROS accumulation. Importantly, we discovered that the expression of SLC25A39 was notably increased in Sorafenib-resistant HCC cells, and silencing of SLC25A39 could sensitize HCC cells to ferroptotic cell death and augment the therapeutic efficacy of Sorafenib against HCC. Overall, our investigation provides comprehensive evidence that SLC25A39 is a critical regulator of ferroptosis evasion and proposes its potential as a therapeutic target to improve the effectiveness of Sorafenib in the treatment of HCC.

**Supplementary Information:**

The online version contains supplementary material available at 10.1186/s12935-025-04151-9.

## Introduction

Mitochondria play crucial roles in controlling numerous cellular processes, including energy production and death [1–3]. Additionally, these organelles are vital in the modulation of reactive oxygen species (ROS) production and thus redox homeostasis [4]. Emerging evidence has linked mitochondrial redox imbalance to multiple age-related diseases, including cancer [5]. The SLC25 carrier family facilitates the transport of metabolites across mitochondrial membranes to control multiple functions of mitochondrial [6]. Recently, SLC25A39 has been revealed to play an essential role in mitochondrial GSH (mGSH) import from the cytoplasm to maintain redox balance and avoid mitochondrial dysfunction [7]. Our bioinformatics analysis showed that SLC25A39 is notably increased in HCC. However, the biological roles of SLC25A39 in human cancers, particularly in hepatocellular carcinoma, remain largely uninvestigated.

Programmed cell death (PCD) is essential for maintaining biological homeostasis and its dysregulation is closely linked to the progression of various human diseases, including cancer [8]. As a new form of PCD, ferroptosis is different from apoptosis, necrosis and autophagy in pathological process [9, 10]. Studies have revealed that diminished ferroptosis is closely linked to various malignancies [11, 12]. Ferroptosis is marked by iron-associated accumulation of lipid ROS [13]. Oxidative stress caused by either destruction of cellular antioxidants or increased ROS production has been closely associated with ferroptosis in several types of diseases [14–17]. However, the relevance of redox state inside mitochondrial and ferroptosis remains largely unknown.

In the current investigation, we systematically examined the clinical significance and biological functions of SLC25A39 in. Our data show that SLC25A39 promotes HCC survival and growth by inhibiting ferroptosis via preventing mitochondrial ROS accumulation through facilitating mitochondrial GSH import. More importantly, we observed that SLC25A39 expression was elevated in Sorafenib resistant HCC cells and its silencing could enhance the anti-cancer activity of sorafenib in HCC treatment, suggesting SLC25A39 as a promising target for improving sorafenib sensitivity in HCC management.

## Materials and methods

### Reagents and HCC cell lines

Sorafenib, ferrostatin-1 (Fer-1), liproxstatin-1 (Lip-1), Z-VAD-FMK (ZVF), Necrostatin-1 (Nec-1) and N-acetylcysteine (NAC) were obtained from Selleck. RSL3 was purchased from Sigma.

Five human HCC cell lines (SNU-449, SNU-423, Hep3B, SNU-354 and HLF) and a normal liver cell line (THLE-2) were maintained in DMEM (Gibco, China) containing 10% FBS (PAN-Seratech, Germany) at 37 °C with 5% CO_2_. All cells underwent authentication via fingerprinting and routinely screened for mycoplasma infection.

### Tissue samples from HCC patients

The paired tumor and adjacent tissue were collected from 248 HCC patients in our hospital (Shaanxi Provincial People’s Hospital). Informed consents have been collected from the participants prior to the study. The study was approved by the Institute’s Research Ethics Committee of the Shaanxi Provincial People’s Hospital and conducted according to the ethical guidelines of the World Medical Association Declaration of Helsinki.

### qRT-PCR

RNA extraction was carried out using TRIzol Reagent (Invitrogen), followed by reverse transcription to cDNA using a cDNA synthesis kit (Takara). Quantitative RT-PCR (qRT-PCR) analysis was conducted utilizing the SYBR Green PCR kit (Roche), with primer sequences listed in Table S1. The results were analyzed using the 2^−ΔΔCt^ method.

### Immunohistochemistry (IHC) staining

IHC staining was performed with an IHC assay kit purchased from Fuzhou Maixin Biotech. Antibodies used in IHC analysis are provided in the Table S2. Images were captured with a microscope from the Olympus company and the staining intensity was scored by multiplying the positive staining percentage score (0, 1, 2, 3, 4 for < 5%, 5–25%, 26–50%, 51–75%, 76–100%) and intensity score (0 for negative, 1 for weak, 2 for moderate and 3 for strong).

### Plasmid construction and cells transfection

Short hairpin RNA (shRNA) was utilized to knockdown SLC25A39 with a pSilence-2.1 vector. For construction of SLC25A39 plasmid, SLC25A39 cDNA was PCR amplified and cloned into a pcDNA3.1 vector. Both shRNA and over-expression plasmid were transfected using lipofectamine 3000 reagent (Invitrogen, USA) 48 h before further investigations. To generate stable SLC25A39 knockdown or overexpression HCC cells, clones were selected using puromycin (4 µg/ml) or G418 (600 µg/ml) for 10 days, respectively. The resistant colonies were expanded and maintained in a lower puromycin (2 µg/ml) or G418 (300 µg/ml). The in vitro knockdown or overexpression efficiency of SLC25A39 was validated by qRT-PCR and western blotting assays. The in vivo knockdown efficiency of SLC25A39 was confirmed by IHC staining assay.

### Cell proliferation assays

The viability of HCC cells was assessed by a CCK-8 kit (Beyotime, China). HCC cells (2000 cells/well) were cultured in 96-well plates and CCK8 reagent was added. The absorbance at 450 nm was detected at indicated time point.

Cell proliferation (longer term) was also assessed by colony formation assay. HCC cells were cultured in 6-well plates at a density of 1000 cells per well for approximately 10 days. Then, the colonies in each well were fixed by 4% paraformaldehyde and subsequently stained with crystal violet. After that, the colonies were washed and numbered.

### Cell cycle and cell death

For evaluations of the cell cycle and cell death of HCC cells with different treatment, a cell cycle detection kit or Annexin V-FITC detection kit purchased from Everbright Biotech was used. The results were analyzed by a flow cytometry.

### Cell migration and invasion

For assessing cell migration, a scratch wound healing assay was employed. HCC cells were maintained in a 6-well plate in serum free medium. Scratches were then made when cells reached to about 80% confluent. Photos were taken at indicated time point after scratching and relative wound closure was subsequently calculated.

For the transwell invasion assay, HCC cells were seeded into 8-µm invasion chambers containing matrigel matrix. After a 48-hour incubation period, the cells that had invaded were fixed with 4% paraformaldehyde and stained with hematoxylin for photographing under an inverted microscope.

### Western blotting

RIPA buffer was used to obtain cell lysates. After centrifugation for 20 min, supernatants containing cell protein were collected and loaded in SDS-PAGE gels for separation by electrophoresis. Then, protein separated in gels was transferred to the PVDF membrane followed by blocking with non-fat milk. After that, primary and secondary antibodies were added. Finally, the membranes were subjected to an enhanced chemiluminescence detection system for protein expression.

### Animal assays

Animal experiments were performed in accordance with the guidelines established by the Institutional Animal Care and Use Committee of our hospital. Control and SLC25A39-silencing SNU-423 cells (3 × 10^6^) were injected orthotopically into the livers (for tumor growth) or through tail veins (for metastasis) to the nude mice (BALB/c, 3-4-week-old, six mice per group). Tumor volume was measured weekly. For in vivo tumor growth assay, the animals were euthanized 4 weeks post the cells injection and their livers were harvested. For in vivo metastasis assay, mice were euthanized 6 weeks after cells injection and metastasis in their lungs was assessed by H༆E staining assay.

To evaluate the concomitant SLC25A39 silencing and Sorafenib treatment on HCC growth, the in vivo subcutaneous xenograft model was constructed by subcutaneously injecting control or SLC25A39 silencing SNU-423 cells to the nude mice. One week after cells injection, Sorafenib (30 mg/kg every day) or vehicle (5% DMSO dissolved in saline) treatment was initiated and administered intragastrically. Three weeks later, the animals were humanely sacrificed for the collection of tumors.

### Lipid peroxidation measurement assay

For lipid peroxidation measurement, the BODIPY-C11 kit (D3861, Thermo Fisher) was utilized. Briefly, 5 µM BODIPY-C11 was added to HCC cells with different treatment and incubated with for 25 min at 37 °C. Washing three times with PBS was then performed and lipid peroxidation level was analyzed with a flow cytometer.

### Transmission electron microscopy

HCC cells grown to 70–80% confluence were collected, washed twice with ice-cold PBS and fixed with 2.5% glutaraldehyde in sodium cacodylate buffer (pH 7.2). Subsequently, the samples were rinsed with 0.1 M sodium cacodylate buffer and incubated with 0.1% tannic acid. After that, the samples were fixed using 1% buffered osmium solution and underwent en bloc staining with 1% uranyl acetate. After dehydration in a graded ethanol series, the samples were infiltrated and embedded, and polymerisation was completed at 62 °C for 48 h. Ultrathin sections were cut, collected on 200-mesh copper grids, stained with uranyl acetate and examined by transmission electron microscopy (FEI, Hillsboro, Oregon, USA).

### Mitochondrial isolation assay

HCC cells were homogenized in ice-cold mitochondrial isolation buffer (Beyotime, #C3601). Cell lysates were centrifuged at 4000 × g (20 min). The supernatant containing mitochondria was washed by PBS and centrifuged again at 11,000× g (15 min).

### Determinations of GSH and GSSG levels

The levels of GSH and its disulfide-oxidized form GSSG were assessed by a commercial kit obtained from the Beyotime Biotechnology. The corresponding GSH or GSSG content was determined according to the standard curve and the ratio of GSH/GSSG was then calculated.

### Total cellular and mitochondrial ROS measurements

For the measurement of total cellular ROS level, a commercial kit obtained from the Beyotime Biotechnology was used when HCC cells were cultured to 60–80% confluency. The imaging was performed with an Olympus confocal microscope.

MitoSOX™ (Invitrogen, USA) red dye was used for assessment of mitochondrial ROS levels when HCC cells were cultured to 60–80% confluency. After a 10-minute incubation at 37 °C, HCC cells were washed and dyed with DAPI. An Olympus confocal microscope was used for photos taken.

### Assessment of mitochondrial oxygen consumption rate (OCR)

Mitochondrial OCR was measured using a Seahorse XFe96 extracellular flux analyser (Agilent). HCC cells were plated into Seahorse XF plates 18 h prior to the assay. After replacing the culture medium with Seahorse XF base medium, plates were incubated 60 min without CO₂. Following incubation, OCR was recorded under basal conditions and oligomycin (1.5 µM), FCCP (0.5 µM) and rotenone (1 µM) were then sequentially injected. OCR was normalized to total protein content.

### Detections of mitochondrial membrane potential and ATP level

Mitochondrial membrane potential was evaluated using JC-1 dye purchased from Beyotime Biotechnology as per the manufacturer’s protocol. The results are expressed as the ratio of aggregate-to-monomer (red-to-green) fluorescence intensity. Cellular ATP level was quantified using a commercial ATP colorimetric/fluorometric assay kit (abcam) in accordance with the manufacturer’s protocol. The results were normalized to the protein concentrations measured in the samples.

### Generation of Sorafenib resistant HCC cells

Sorafenib-resistant SNU-423 and HLF cell lines were generated by culturing cells with increasing doses of sorafenib (2 µM for 4 weeks, 4 µM for 4 weeks, 8 µM for 8 weeks). The established sorafenib-resistant cells were sustained by 10 µM sorafenib.

### Detections of serum AST and ALT levels

AST and ALT levels in cell culture medium were measured using commercial kits purchased from Nanjing Jiancheng Bioengineering Institute (#C010-2-1 for AST, #C009-2-1 for ALT). In brief, 5 µL of cell culture medium were mixed with 20 µL buffer 1 and incubated at 37 °C for 30 min. After that, 20 µL buffer 2 was added and incubated again at 37 °C for 30 min. The reaction was stopped by adding 200 µL 0.4 mol/L NaOH. Absorbance was recorded using a plate reader at 505 nm.

### Statistical analysis

To determine the difference between two experimental groups, the student’s t-test was used. To determine the difference among multiple experimental groups, one-way ANOVA followed by Dunnett’s or Tukey’s post-hoc test was employed. For patients’ survival analysis, Kaplan–Meier curves analysis was employed to assess statistical significance. Correlations were analyzed using Pearson correlation coefficients. All quantitative experimental data are expressed as mean ± SD. *, P-value < 0.05. ns, not statistical significant.

## Results

### SLC25A39 is upregulated and predicts worse patients’ survival in HCC

**Fig. 1:**
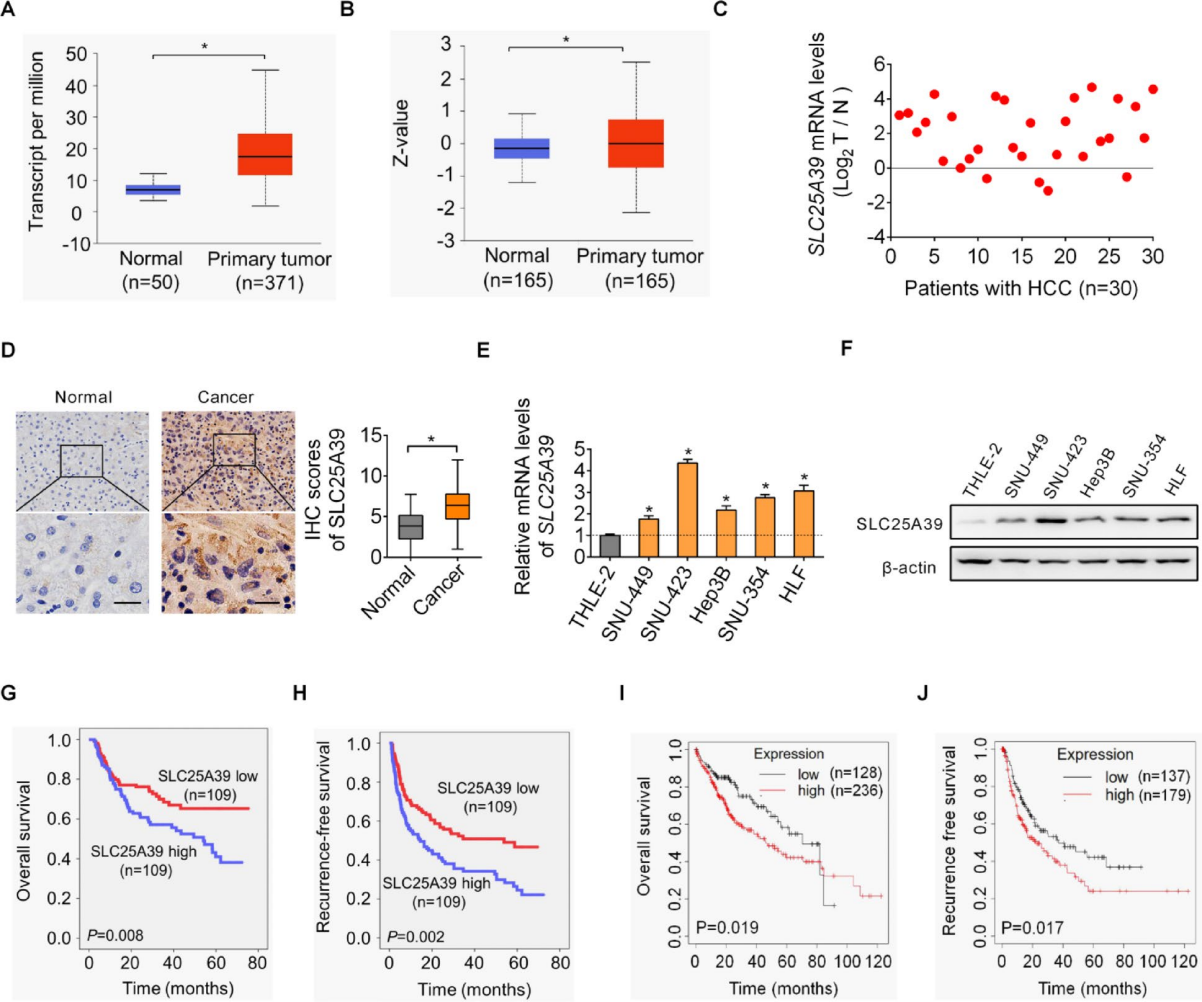
SLC25A39 is upregulated and predicts worse patients’ survival in HCC. (**A** and **B**) The expressions of SLC25A39 were evaluated in the online UALCAN database in HCC at either mRNA (**A**) or protein (**B**) level. (**C** and **D**) SLC25A39 expression was determined in a cohort of 248 pairs of HCC and corresponding adjacent non-tumor tissues. Among them, 30 pairs were used for mRNA expression determination by qRT-PCR analysis (**C**), and the other 218 pairs were used for protein expression determination by immunohistochemistry staining assay (**D**, Scale bars = 10 μm). (**E** and **F**) SLC25A39 expression was determined by qRT-PCR (**E**) and western blotting (**F**) assays in cell lines of HCC and normal liver. (**G** and **H**) Overall (**G**) and recurrence-free (**H**) survivals were compared between HCC patients exhibiting high or low SLC25A39 protein expression at based on our immunohistochemistry staining results in tumor tissue samples from 218 patients. (**I** and **J**) Overall survival (**I**) and recurrence-free survival (**J**) of HCC patients were also evaluated utilizing the online Kaplan-Meier plotter database according to the expressions of SLC25A39

The expression levels of SLC25A39 in HCC were firstly analyzed using the online UALCAN database [18]. The analysis revealed significantly elevated levels of SLC25A39 at both mRNA and protein levels in tumor tissues compared to normal liver tissues (Fig. 1A and B). To validate this, SLC25A39 expression was further analyzed in a cohort of 248 pairs (30 for mRNA expression; 218 for protein expression) of HCC and adjacent non-tumor tissues using qRT-PCR and immunohistochemistry (IHC) staining assays. Consistent with the findings from the online UALCAN database, higher levels of SLC25A39 were also observed in tumor tissues of HCC relative to adjacent non-tumor tissues (Fig. 1C and D), with a 67.6% positive rate in adjacent non-tumor tissues versus a 97.1% positive IHC staining rate in HCC tissues. In addition, in agreement with HCC tissues, the expression of SLC25A39 was also increased in HCC cell lines in comparison with normal hepatocyte (Fig. 1E and F), as evaluated by qRT-PCR and western blot analyses. Evaluation of the subcellular localization of SLC25A39 in HCC cells by immunofluorescence (IF) staining assay showed clear co-localization of SLC25A39 with the mitochondrial outer membrane protein TOM20 (Translocase of Outer Mitochondrial Membrane 20) in both SNU-423 and HLF cells expressing high levels of SLC25A39 (Fig. S1A), suggesting that SLC25A39 functions primarily by localizing to mitochondria in HCC cells. We also analyzed the expressions and clinical implications of SLC25A39 in Sangerbox 3.0 database and found that, in line with HCC, upregulation of SLC25A39 was found in most human cancer types (Fig. S1B) and its upregulation was closely associated with poorer patients’ survival (Fig. S1C). Moreover, prognostic analysis of SLC25A39 was conducted based on the IHC staining results in tumor samples from 218 HCC patients. HCC patients expressing high SLC25A39 exhibited significantly worse survival rates compared to those expressing low SLC25A39 (Fig. 1G and H). Besides, the expression level of SLC25A39 was positively associated with the size of tumor (Table S3). Consistent results were also obtained using the online survival analysis tool Kaplan-Meier Plotter (Fig. 1I and J).

### Knockdown of SLC25A39 suppressed the HCC growth by impairing cell survival

**Fig. 2:**
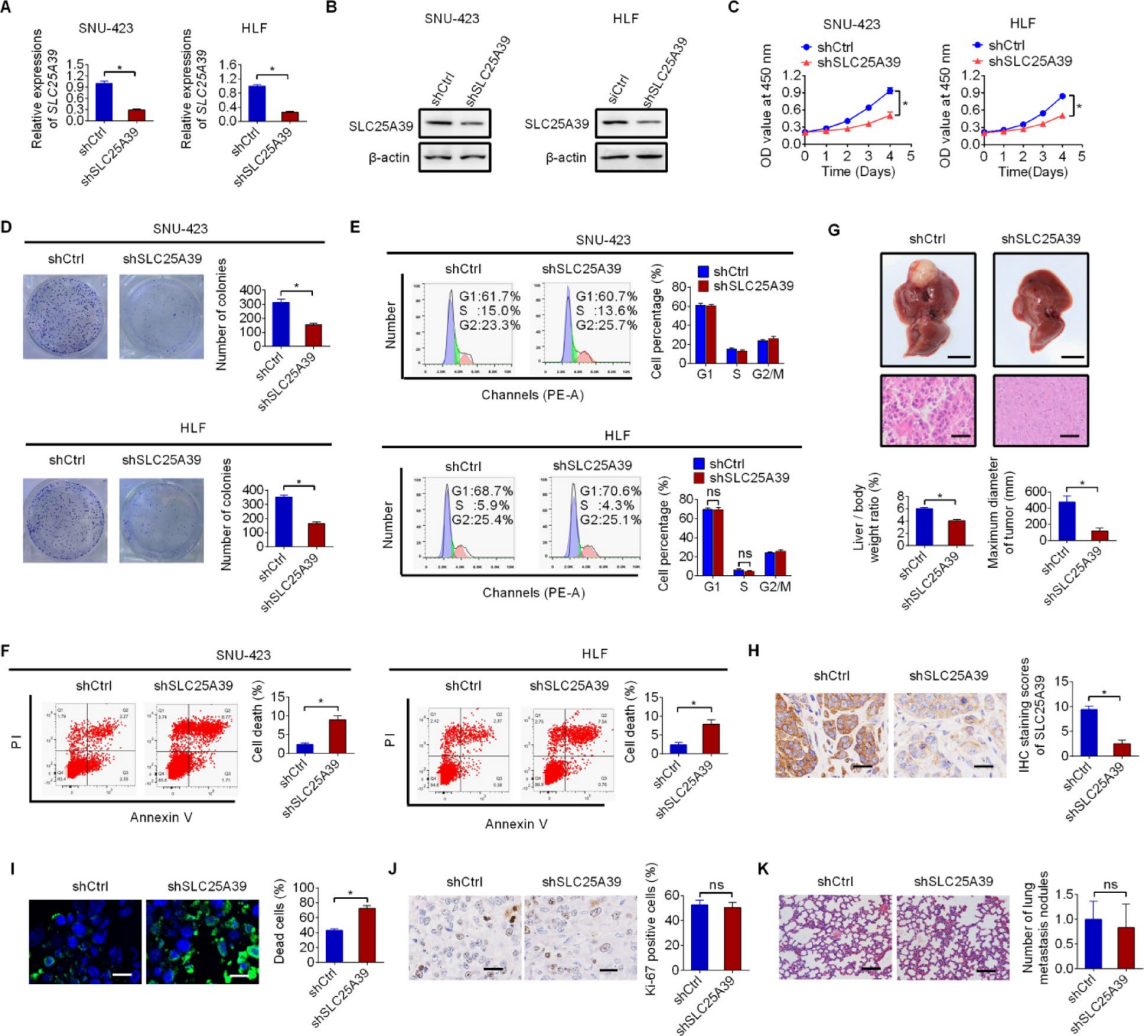
Knockdown of SLC25A39 suppressed the HCC growth by impairing cell survival. (**A** and **B**) SLC25A39 knockdown was verified by qRT-PCR (**A**) and western blotting (**B**) in SNU-423 and HLF cell lines with or without SLC25A39 silencing. (**C** and **D**) Cell Counting Kit-8 (CCK-8, **C**) and colony formation (**D**) assays were used to assess cell proliferation in SNU-423 and HLF cell lines with or without SLC25A39 silencing. (**E** and **F**) Cell cycle (**E**) and death (**F**) were assessed in SNU-423 and HLF cell lines with or without SLC25A39 silencing. (**G**) Tumor formation (Upper panel, gross liver images, Scale bars = 5 mm; Middle panel, Hematoxylin & Eosin staining images, Scale bars = 25 μm; Bottom panel, liver/body weight (LW/BW) ratio and maximum diameter of tumor) were evaluated in the nude mice orthotopically injected with SLC25A39 silencing or control SNU-423 cells. (**H** and **I**) Immunohistochemical staining assay for SLC25A39 expression (**H**, Scale bars = 25 μm) and TUNEL staining assay (**I**, Scale bars = 10 μm) were conducted in xenograft tumor tissues developed from SLC25A39 silencing or control SNU-423 cells. (**J**) Immunohistochemical staining assay for Ki-67 expression was conducted in SLC25A39 silencing or control xenograft tumor tissues (Scale bars = 25 μm). (**K**) Metastatic foci was quantified in the lungs of control and SLC25A39 silencing (Scale bars = 50 μm)

To confirm the role of SLC25A39 in HCC, SLC25A39 was knocked-down in SLC25A39 high expressing SNU-423 and HLF cell lines (Fig. 2A and B). Cell proliferation assays (CCK-8 and colony formation) showed that SLC25A39 silencing markedly abolished both the survival and growth of HCC cells (Fig. 2C and D), while wound healing and matrigel-coated transwell assays revealed no notable changes in cell metastasis after SLC25A39 silencing (Fig. S2A and S2B). Suppressed HCC survival and growth could be due to repressed cell cycle and/or enhanced cell death. Accordingly, the effect of SLC25A39 silencing on cell cycle and cell death were evaluated. Cell cycle assays showed no significant change in the percentage of cell cycle distribution, while cell death was significantly increased when SLC25A39 was knocked-down (Fig. 2E and F), indicating that SLC25A39 promotes HCC growth by impairing cell survival.

Next, the contribution of SLC25A39 to HCC growth and metastasis in vivo was validated in nude mouse models by injecting HCC cells orthotopically into the livers (for tumor growth) or through tail veins (for tumor metastasis) (six mice per group). As shown in Fig. 2G, tumor formation in the liver, liver/body weight (LW/BW) ratio and maximum diameter of tumor were markedly reduced when SLC25A39 was silenced. Immunohistochemical staining assay confirmed decreased SLC25A39 expression in tumors derived from SLC25A39-silenced SNU-423 cells (Fig. 2H). TUNEL staining assay also indicated significantly increased cell death in tumor tissues after SLC25A39 silencing (Fig. 2I), while the percentage of proliferating cells was comparable in tumor tissues developed from SLC25A39 silencing or control SNU-423 cells (Fig. 2J). However, the number of lung metastatic foci in control and SLC25A39-silenced nude mice was comparable (Fig. 2K). Together, these results suggest that knockdown of SLC25A39 suppressed HCC growth through impairing cell survival.

### SLC25A39 overexpression promoted HCC growth

**Fig. 3:**
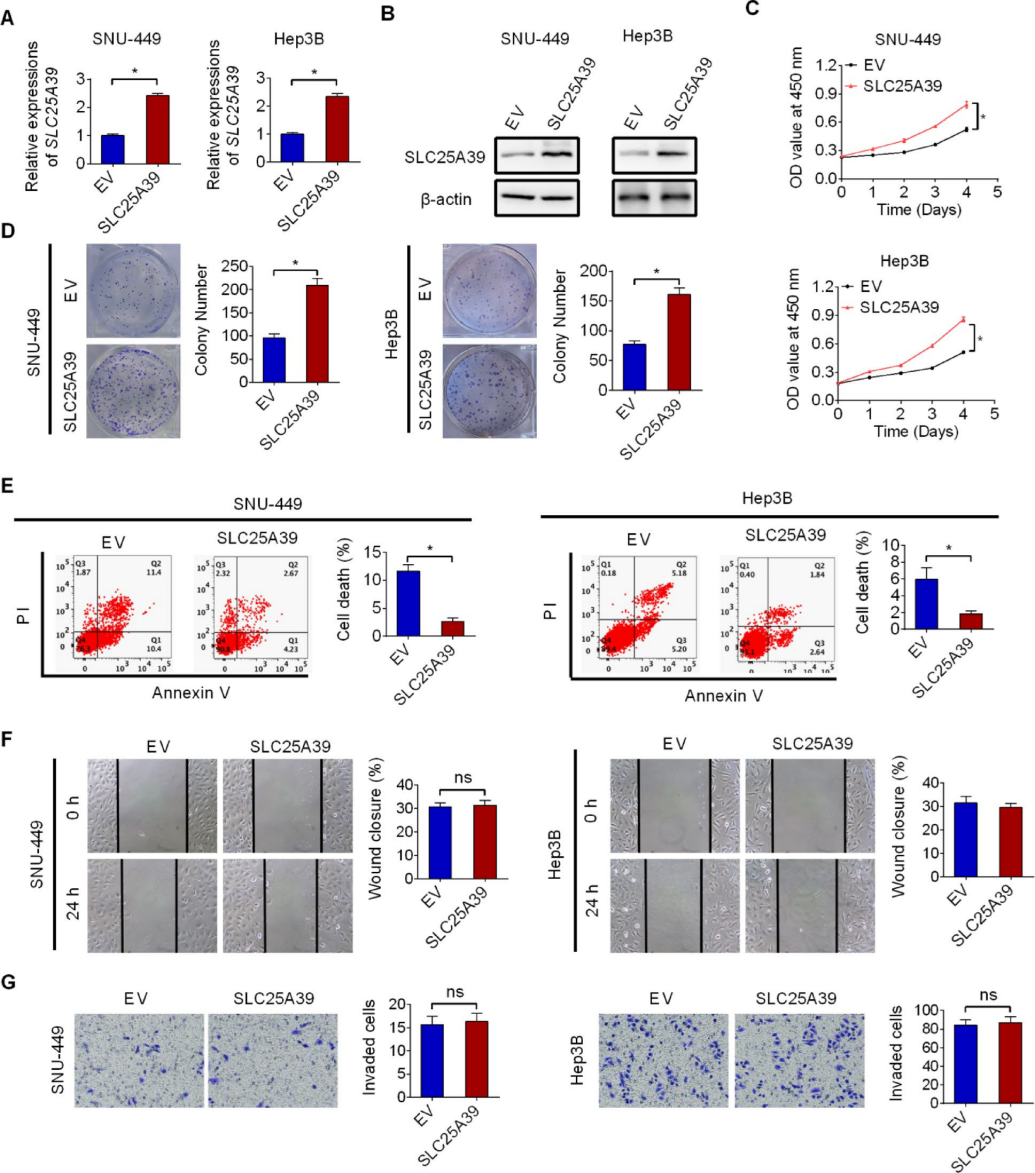
SLC25A39 overexpression promoted HCC growth. (**A** and **B**) SLC25A39 overexpression was verified by qRT-PCR (**A**) and western blotting (**B**) assays in SNU-449 and Hep3B cell lines with forced SLC25A39 expression. (**C** and **D**) CCK-8 (**C**) and colony formation (**D**) assays were used to assess cell proliferation in SNU-449 and Hep3B cell lines with forced SLC25A39 expression. (**E**) Cell death analysis was conducted in SNU-449 and Hep3B cell lines with forced SLC25A39 expression. (**F** and **G**) Wound healing (**F**) and matrigel-coated transwell invasion (**G**) assays were used to assess cell metastasis ability in SNU-449 and Hep3B cell lines with forced SLC25A39 expression

To further confirm the role of SLC25A39 in HCC growth, SLC25A39 was overexpressed in SLC25A39 low expressing HCC cell lines of SNU-449 and Hep3B (Fig. 3A and B). Forced SLC25A39 expression significantly enhanced both the survival and growth of HCC cells (Fig. 3C and D). Cell death analysis indicated markedly suppressed cell death (Fig. 3E), while no significant changes in cell migration (Fig. 3F) and invasion (Fig. 3G) were observed in SNU-449 and Hep3B cells upon SLC25A39 overexpression. To provide more evidence for the oncogenic role of SLC25A39 in HCC, the effects of SLC25A39 overexpression were also evaluated in a non-malignant hepatocyte line THLE-2. Similar to the effects observed in HCC cell lines, forced SLC25A39 expression also significantly enhanced cell viability and colony formation ability in THLE-2 cells (Fig. S3A-S3D). These results provide further support for the oncogenic functions of SLC25A39 in the promotion of HCC growth.

### SLC25A39 promotes HCC cell survival by suppressing ferroptosis

**Fig. 4:**
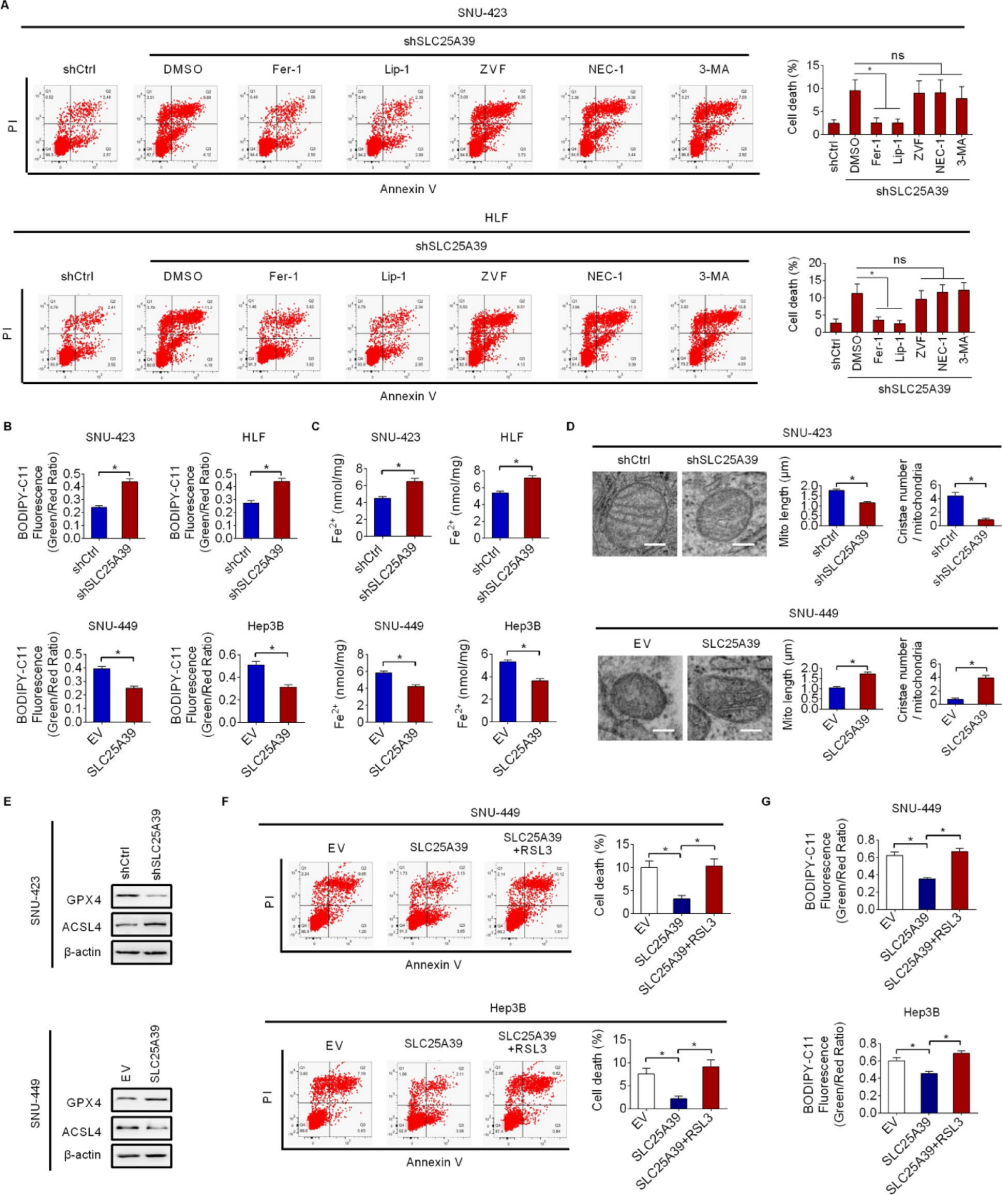
SLC25A39 promotes HCC cell survival by suppressing ferroptosis. (**A**) SLC25A39 knockdown SNU-423 and HLF cells were subjected to various inhibitors targeting distinct pathways of cell death (Fer-1 and Lip-1 were used to inhibit ferroptosis; ZVF was used to suppress apoptosis; Nec-1 was used to inhibit necroptosis; 3-MA was used to suppress autophagy). (**B**) Lipid peroxidation in indicated HCC cells with SLC25A39 silencing or overexpression was evaluated. (**C**) The levels of Fe^2+^ were determined in indicated HCC cells with SLC25A39 silencing or overexpression. (**D**) The morphology of mitochondria was evaluated using a transmission electron microscopy (TEM) analysis in indicated HCC cells with SLC25A39 silencing or overexpression (Scale bars = 0.5 μm). (**E**) The expressions of key ferroptosis modulators GPX4 and ACSL4 were determined by western blotting in indicated HCC cells with SLC25A39 silencing or overexpression. (**F** and **G**) Cell death (**F**) and lipid peroxidation (**G**) in SNU-423 and Hep3B cells were determined

Mitochondria have been well documented as the central regulators of different types of cell death. To explore the specific type of cell death that was regulated by SLC25A39, HCC cells were treated with inhibitors targeting different cell death pathways, including ferroptosis inhibitors Fer-1 or Lip-1, apoptosis inhibition by Z-VAD-FMK, necroptosis inhibition by Necrostatin-1, and autophagy inhibition by 3-Methyladenine, respectively. The results showed that SLC25A39 knockdown-induced cell death was markedly attenuated only by the application of ferrostatin-1 (Fer-1) or liproxstatin-1 (Lip-1), both of which inhibit ferroptosis (Fig. 4A), suggesting that SLC25A39 knockdown promotes HCC cell death by inducing ferroptosis. To provide further support, we determined the effect of SLC25A39 silencing on lipid peroxidation, a hallmark of ferroptosis, using C^11^-BODIPY581/591 probes. The results showed that SLC25A39 silencing also markedly increased lipid peroxidation, whereas forced SLC25A39 expression markedly decreased lipid peroxidation in HCC cells (Fig. 4B). In agreement, the intracellular levels of Fe^2+^ were also elevated upon SLC25A39 silencing and reduced upon SLC25A39 overexpression (Fig. 4C). Additionally, transmission electron microscopy (TEM) analysis showed a significant mitochondrial damage marked by enhanced mitochondrial contraction and disrupted cristae structure upon SLC25A39 silencing, whereas the opposite effects were observed upon SLC25A39 overexpression (Fig. 4D). In agreement with these observations, SLC25A39 silencing resulted in clearly decreased GPX4 expression and increased ACSL4 expression in SNU-423 cells. By contrast, increased GPX4 and decreased ACSL4 expression were observed upon SLC25A39 upregulation in SNU-449 cells (Fig. 4E). Moreover, we found that the decreased cell death and lipid peroxidation caused by SLC25A39 overexpression were significantly reversed by RSL3 treatment (Fig. 4F and G). Together, the above data strongly suggest that SLC25A39 facilitates HCC cell survival mainly by suppressing ferroptosis.

### SLC25A39 inhibits ferroptosis by preventing mitochondrial ROS accumulation via facilitating mitochondrial GSH import

**Fig. 5:**
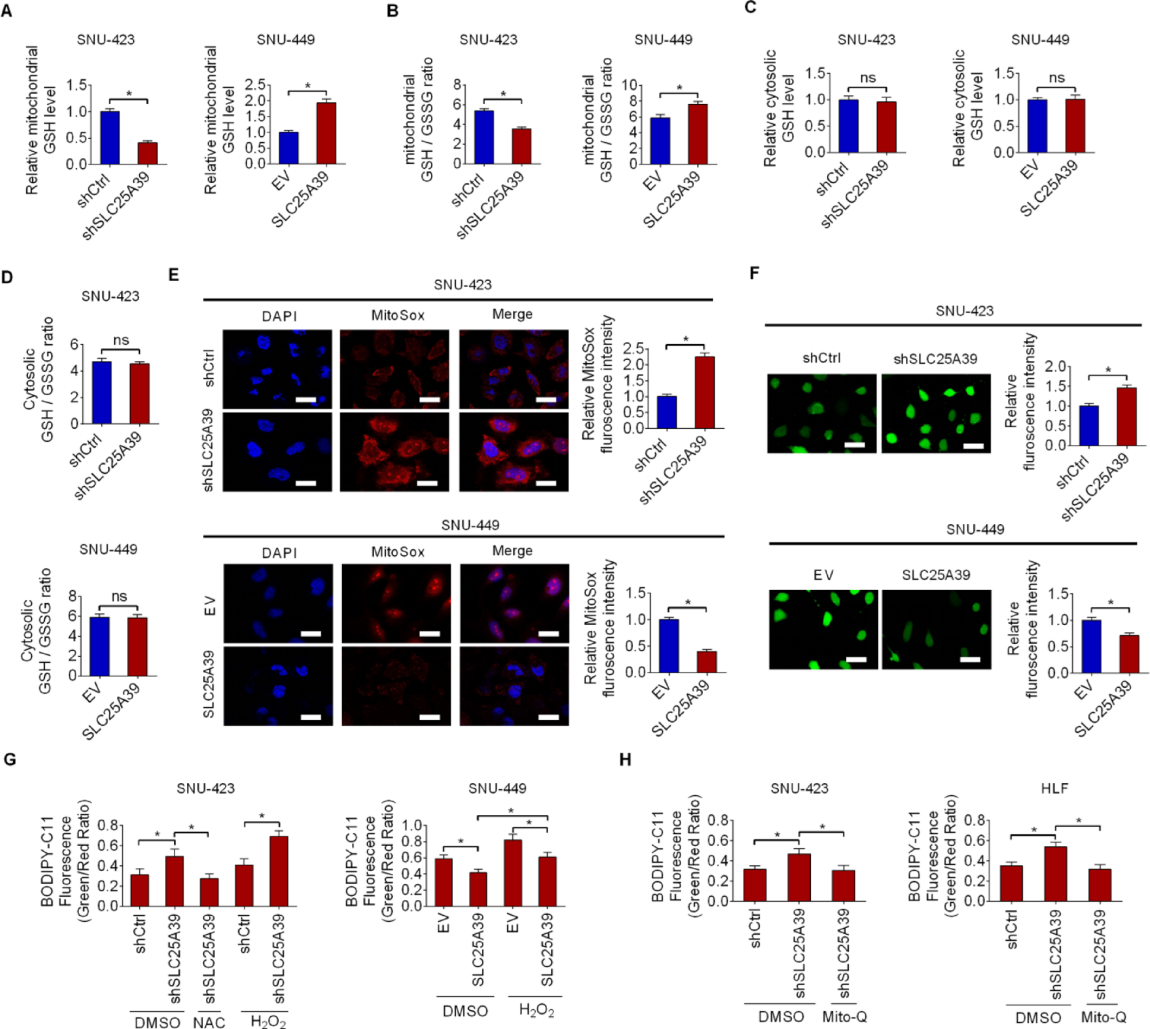
SLC25A39 inhibits ferroptosis by preventing mitochondrial ROS accumulation via facilitating mitochondrial GSH import. (**A**) The content of GSH was determined in isolated mitochondria from SLC25A39 silencing or overexpression HCC cells. (**B**) GSH/GSSG ratio was analyzed in isolated mitochondria from SLC25A39 silencing or overexpression HCC cells. (**C** and **D**) The levels of GSH (**C**) and GSH/GSSG ratio (**D**) were determined in the whole cell extract from SLC25A39 silencing or overexpression HCC cells. (**E** and **F**) Mitochondrial fraction or total cellular ROS levels were determined in SLC25A39 silencing or overexpression HCC cells (Scale bars = 10 μm). (**G**) Lipid peroxidation was assessed in SLC25A39 silencing or overexpressing HCC cells treated with NAC (20 mM for 12 h) or H_2_O_2_ (100 µM for 12 h). (**H**) Lipid peroxidation was assessed in SLC25A39 silencing SNU-423 and HLF cells treated with Mito-Q (1 μm) (Scale bars = 10 μm)

Given the critical role of SLC25A39 in mitochondrial GSH import [7], the contents of GSH and GSSG were thus determined in isolated mitochondria from HCC cells after SLC25A39 silencing or overexpression. Both GSH content and GSH/GSSG ratio, an indicator of cellular oxidative stress, decreased significantly in mitochondria when SLC25A39 was silenced and increased when SLC25A39 was overexpressed (Fig. 5A and B). Unlike in the mitochondria, the levels of GSH and ratio of GSH/GSSG in the whole cell extract were not significantly altered when SLC25A39 was silenced or overexpressed in HCC cells (Fig. 5C and D), indicating a specific regulation of SLC25A39 in mitochondrial GSH level in HCC cells. Subsequently, mitochondrial and total cellular ROS levels were determined after silencing or overexpression of SLC25A39. As shown in Fig. 5E, silencing of SLC25A39 led to abnormally high accumulation of mitochondrial ROS, whereas overexpression of SLC25A39 led to decreased mitochondrial ROS. In line with this, a similar effect on total cellular ROS levels was obtained upon SLC25A39 knocking-down or overexpressing (Fig. 5F). Next, the involvement of ROS in SLC25A39-regulated ferroptosis was explored. The results showed that the induction of ferroptosis by SLC25A39 silencing was significantly diminished when intracellular ROS were scavenged by N-acetylcysteine (NAC) treatment, while the suppression of ferroptosis by SLC25A39 overexpression was counteracted by the increase in intracellular ROS levels following hydrogen peroxide (H_2_O_2_) treatment (Fig. 5G). Meanwhile, the results indicated that SLC25A39 silencing also increased intracellular oxidation with elevated ROS, while upregulation of SLC25A39 reduced intracellular oxidation despite the increased ROS (Fig. 5G). Treatment with the mitochondria-targeted antioxidant Mito-Q further confirmed the above results (Fig. 5H). These results suggest that SLC25A39 inhibits ferroptosis by preventing mitochondrial ROS accumulation via facilitating mitochondrial GSH import.

Given that GSH metabolism is central not only to ferroptosis but also to metabolic rewiring, the impact of SLC25A39 upregulation on mitochondrial metabolism was also determined. The results showed that SLC25A39 silencing significantly decreased mitochondrial oxygen consumption rate (OCR), membrane potential and ATP production, while its overexpression had the opposite effects (Fig. S4B-S4D). These data suggest that SLC25A39 upregulation not only suppresses ferroptosis but also enhances mitochondrial metabolism in HCC.

### SLC25A39 Silencing enhanced the anti-cancer activity of Sorafenib

**Fig. 6:**
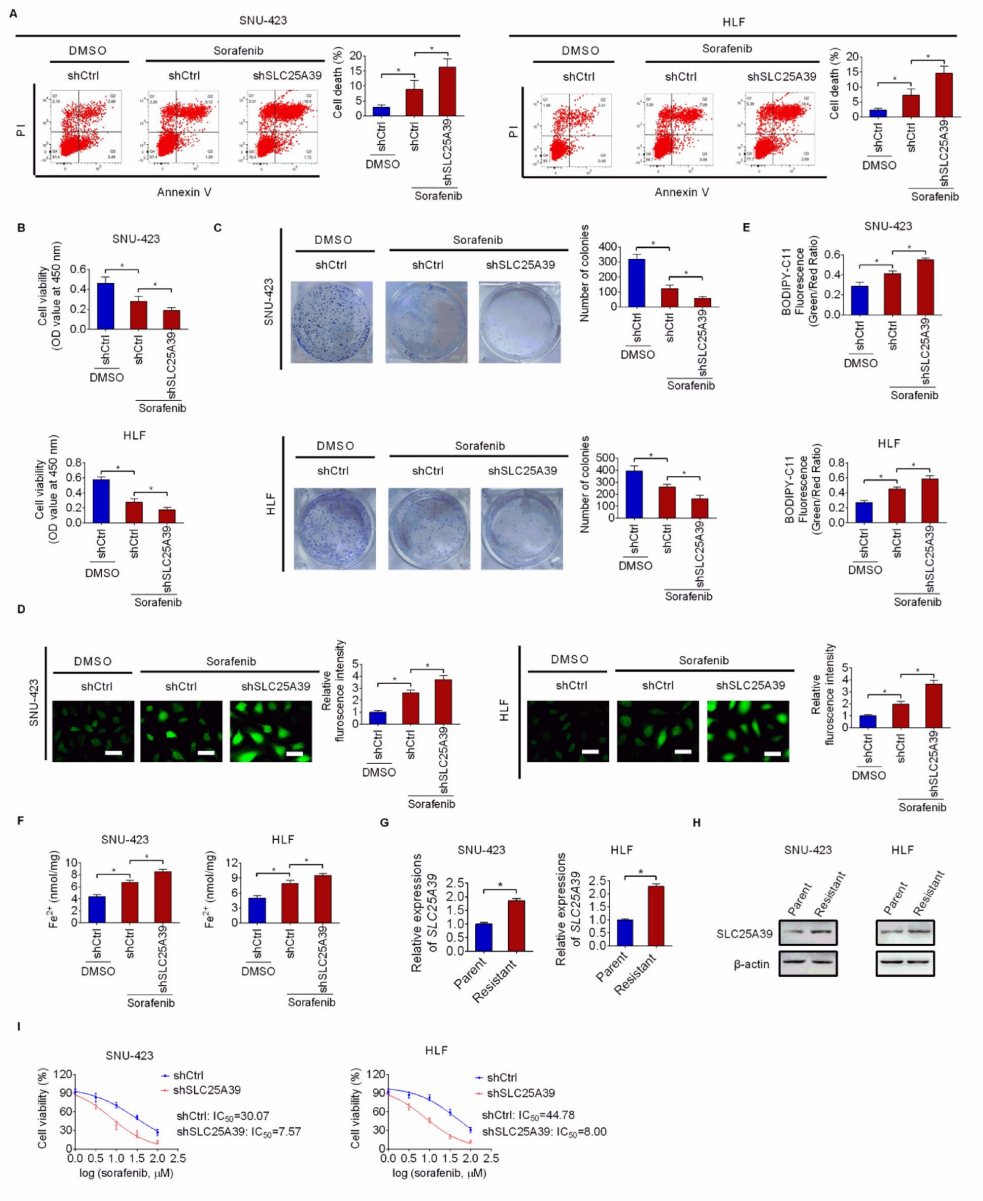
SLC25A39 silencing enhanced the anti-cancer activity of sorafenib in vitro. (**A**) Cell death was examined in SNU-423 and HLF cells upon Sorafenib (10 µM) treatment. (**B**) Cell viability was examined in HCC cells upon Sorafenib (10 µM) treatment. (**C**) Colony formation abilities of HCC cells upon Sorafenib (5 µM) treatment. (**D**-**F**) The intracellular levels of ROS (**D**, Scale bars = 10 μm), lipid peroxidation (**E**) and content of Fe2 ^+^ (**F**) were measured in HCC cells treated with Sorafenib (10 µM). with Sorafenib (10 µM). (**G** and **H**) SLC25A39 expression in Sorafenib-resistant or -sensitive SNU-423 and HLF cells were evaluated by qRT-PCR and Western blotting. (**I**) The effect of SLC25A39 silencing on the sensitivity of sorafenib-resistant SNU-423 and HLF cells to sorafenib treatment was evaluated by IC50 analysis

Sorafenib, a multiple kinases inhibitor, has been approved as a first-line treatment in HCC [19]. However, the effectiveness of sorafenib has been unsatisfactory. Considering that sorafenib has been shown to induce ferroptosis [20], we examined whether silencing of SLC25A39 could enhance the anti-tumor effect of sorafenib in HCC. As shown in Fig. 6A, SLC25A39 silencing markedly aggravated HCC cell death caused by sorafenib treatment. Meanwhile, the suppressive effects of sorafenib on cell viability and colony formation of HCC cells were also obviously enhanced by SLC25A39 silencing (Fig. 6B and C). Expectedly, SLC25A39 silencing also clearly enhanced sorafenib-induced ferroptosis in SNU-423 cells, as evidenced by increased levels of ROS, lipid peroxidation and Fe2 ^+^ (Fig. 6D and F).

Subsequently, sorafenib-resistant cells were developed in SNU-423 and HLF cells by gradually increasing the concentration of sorafenib in culture medium and their resistance to sorafenib was verified by CCK-8 and flow cytometry assays, showing that both cell lines displayed markedly increased sorafenib IC50 and resistance in comparison to their parental counterparts (Fig. S5A-5B). qRT-PCR and Western blotting analysis showed that SLC25A39 expression was clearly increased in Sorafenib-resistant SNU-423 and HLF cells (Fig. 6G and H). Additionally, SLC25A39 silencing in sorafenib-resistant SNU-423 and HLF cells clearly enhanced their sensitivity to sorafenib treatment (Fig. 6I). Moreover, we found that the levels of ALT (alanine aminotransferase) and AST (aspartate aminotransferase) in the cell culture medium of THLE2 cells (normal hepatocytes) were not significantly changed by SLC25A39 silencing (Fig. S5C and S5D), implying that SLC25A39 knockdown has no notable hepatocellular toxicity. These results suggest that SLC25A39 plays a crucial role in Sorafenib resistance and its silencing could enhance the anti-cancer activity of sorafenib in HCC.

**Fig. 7:**
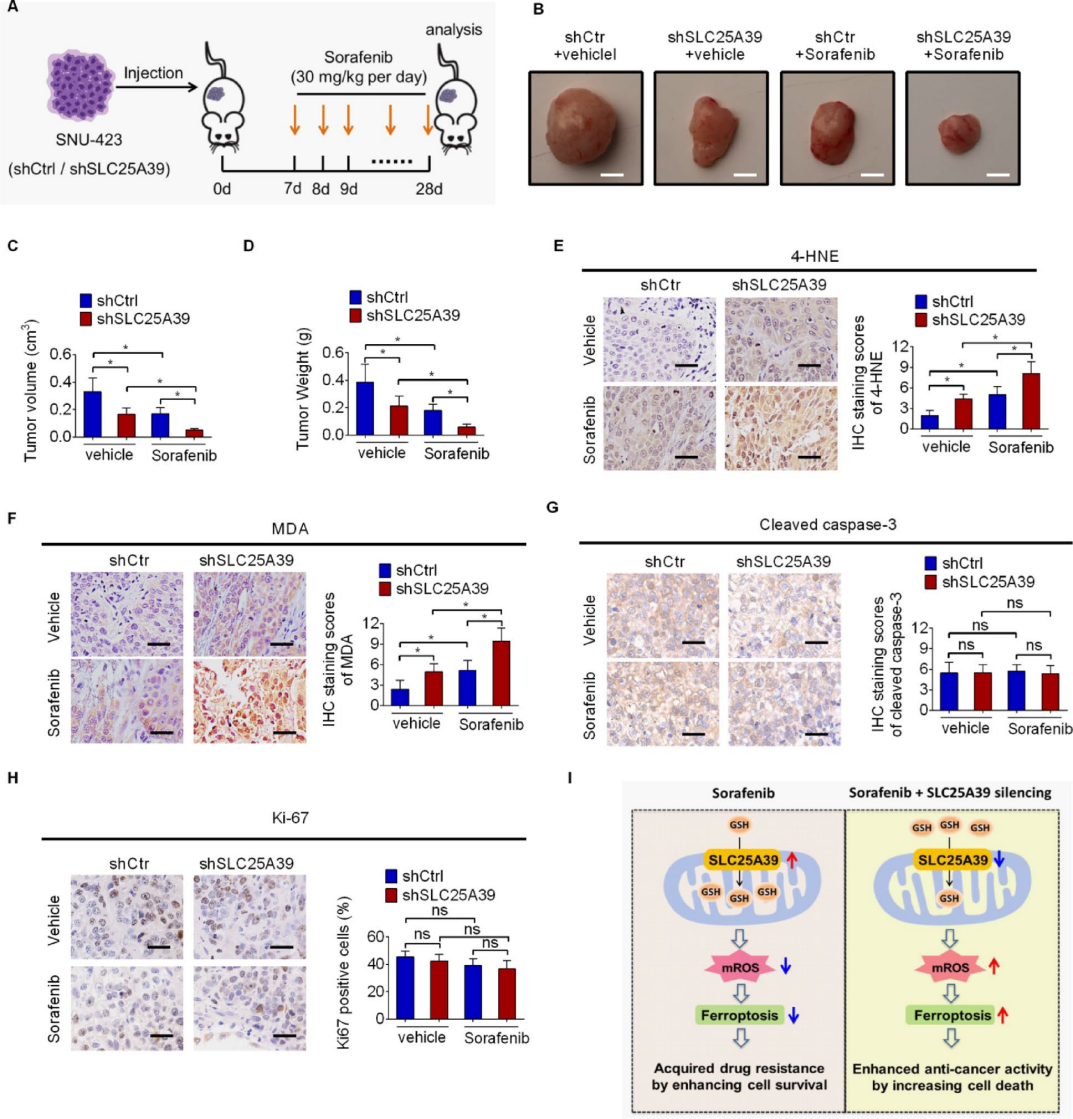
SLC25A39 silencing enhanced the anti-cancer activity of sorafenib in vivo. (**A**) Schematic description of the animal experimental design. (**B**-**D**) Representative photos (**B**, Scale bars = 200 μm), size (**C**) and weight (**D**) of tumors from the nude mice with different treatments indicated were shown (shCtrl + vehicle, shSLC25A39 + vehicle, shCtrl + Sorafenib, shSLC25A39 + Sorafenib). (**E**-**H**) Representative immunochemical images of Ki-67, 4-HNE, MDA and cleaved caspase-3 in xenograft tumors from the nude mice with indicated treatments (shCtrl + vehicle, shSLC25A39 + vehicle, shCtrl + Sorafenib, shSLC25A39 + Sorafenib; Sorafenib, 30 mg/kg every day for 3 weeks; vehicle, 5% DMSO dissolved in saline) (Scale bars = 25 μm). (**I**) Working model shows that SLC25A39 promotes HCC cell survival and Sorafenib resistance by inhibiting mitochondrial oxidative stress-induced ferroptosis

We next sought to determine the impact of concomitant SLC25A39 silencing and Sorafenib treatment on HCC tumor growth in vivo. To test this, the control or SLC25A39 silencing SNU-423 cells were subcutaneously injected into the nude mice. One week post cells injection, the mice were treated with or without Sorafenib (Fig. 7A). Evaluation of tumor growth revealed an obvious suppressive-effect of Sorafenib treatment on HCC growth. Notably, SLC25A39 silencing markedly enhanced the efficacy of Sorafenib treatment in vivo, as evidenced by the fact that the tumors harvested from mice treated with combination of Sorafenib and SLC25A39 silencing were significantly smaller and lighter than those treated with Sorafenib or SLC25A39 silencing alone (Fig. 7B and D). Furthermore, the levels of 4-HNE and MDA, two widely used indicators of lipid peroxidation, were significantly elevated following the combination treatment of Sorafenib and SLC25A39 (Fig. 7E and F). However, the levels of cleaved caspase-3 (cell apoptotic marker) and Ki-67 (cell proliferation marker) showed no significant change upon the combination treatment of Sorafenib and SLC25A39 silencing (Fig. 7G-H). In agreement with the above results, we found that either SLC25A39 silencing or ferroptosis induction by Erastin treatment markedly suppressed tumor growth in Sorafenib-resistant SNU-423 cell-derived HCC mouse models, and their combination exhibited better therapeutic efficacy than either treatment alone (Fig. S6A-S6D). Together, these results indicate that silencing of SLC25A39 sensitizes HCC cells to ferroptotic death and enhances the anti-cancer activity of sorafenib in HCC.

## Discussion

Mitochondria serve as a primary site for the generation of reactive oxygen species (ROS), which contribute to redox imbalance during malignant transformation and the process of hepatic carcinogenesis [21]. A few years ago, SLC25A39, belonging to the SLC25A family, was identified as a critical transporter for mitochondrial glutathione uptake [7], suggesting a critical role for SLC25A39 in redox homeostasis regulation. However, the role of SLC25A39 has rarely been studied in human cancer. In our current study, SLC25A39 expression was shown to be significantly increased in HCC and its upregulation was closely correlated with poor patients’ outcomes in HCC. Additionally, our pan-cancer analysis using the online Sangerbox 3.0 database indicated that the expressions of SLC25A39 were upregulated in most human cancer types and its upregulation was closely associated with poorer patients’ survival in multiple cancer types. Consistent with our findings, the SLC25A39 expression has been reported to be upregulated in pancreatic ductal adenocarcinoma (PDAC), which was significantly related to the prognosis of PDAC [22]. Together, these studies suggest that SLC25A39 upregulation is a frequent event in various human cancers, implying that SLC25A39 may contribute to the carcinogenesis of human cancer.

Functional explorations revealed a crucial oncogenic role of SLC25A39, as its knockdown inhibited in vitro HCC cell viability and colony formation ability without significant influence on cell migration and invasion, while its overexpression promoted HCC cell viability and colony formation. These results were consistently confirmed in vivo. In addition, we found that SLC25A39 promotes HCC growth by suppressing cell death. Notably, we found that SLC25A39 suppresses HCC cell death by decreasing the levels of ferroptosis, which has been characterized by iron-associated lipid peroxidation and proposed as a strategy for the treatment of cancer [23, 24]. These data highlight a crucial role for SLC25A39 in ferroptosis evasion in HCC cells and provide a strategy for cancer therapy.

GSH is a non-enzymatic antioxidant to overcome oxidative stress [25]. It has been demonstrated that GSH functions as a crucial ferroptosis suppressor and its deficiency could promote overwhelming lipid-ROS generation that ultimately leads to ferroptosis [26, 27]. Although being produced in the cytoplasm by two step enzymes GCLC/GCLM and GSS [28], GSH is also abundant in mitochondria and other organelles. Therefore, the uptake of GSH in mitochondria depends on the transportation by SLC25A39. As the major source of ROS, mitochondria require enough GSH to prevent oxidative damage. However, the changes in mitochondrial GSH level remain largely unknown in human cancer cells. Our data show that upregulation of SLC25A39 increased mitochondrial but not total cellular levels of GSH in HCC cells. In line with our observations, it has been reported that loss of SLC25A39 reduced mitochondrial GSH abundance without affecting total cellular GSH levels in HEK-293T and Hela cells [7]. Additionally, GSH/GSSG, which reflects the level of cellular oxidative stress, was markedly increased by SLC25A39 in HCC cells, suggesting that SLC25A39 contributes to the antioxidant defense in HCC cells. In keeping with this, the levels of mitochondrial ROS were significantly decreased by SLC25A39, while the levels of total cellular ROS were not changed significantly. These findings further support the necessary role for SLC25A39 in mitochondrial GSH import under both physiological and pathological conditions across various cell types. However, although the study by Ying Wang et al.^7^ provided strong evidence that SLC25A39 is a mitochondrial carrier required for GSH import, the mechanism by which SLC25A39 regulates glutathione transport across the mitochondrial membrane remains unclear. Further structural studies are still required to determine the biophysical details of mitochondrial GSH import.

Sorafenib was the first approved drug for patients with advanced HCC [29, 30]. However, the efficacy of sorafenib is often limited and still unsatisfactory. Considering that sorafenib has been reported to induce ferroptosis, we therefore explored whether SLC25A39 silencing could enhance the tumor-suppressive activity of sorafenib in HCC and found that SLC25A39 silencing markedly enhanced sorafenib-induced ferroptosis in HCC cells, indicating that SLC25A39 silencing sensitizes HCC cells to ferroptotic death. Furthermore, we found that SLC25A39 expression was significantly induced by Sorafenib treatment, indicating that SLC25A39 may contribute to the development of Sorafenib resistance. Together, these findings hold potentials for SLC25A39 as promising target to improve the efficacy of sorafenib treatment in HCC patients.

## Conclusions

In summary, our data showed that SLC25A39 serves as a pivotal promoter of HCC by inhibiting ferroptosis via preventing mitochondrial ROS accumulation through facilitating mitochondrial GSH import. More importantly, SLC25A39 silencing markedly enhanced sorafenib-induced ferroptosis in HCC cells, strongly implying SLC25A39 as a potential target for increasing the benefit from sorafenib treatment in HCC (Fig. 7I).

## Supplementary Information

Below is the link to the electronic supplementary material.

Supplementary Material 1

## Data Availability

No datasets were generated or analysed during the current study.

## Abbreviations

SLC25A46: Solute carrier family 25 member 46
HCC: Hepatocellular carcinoma
GSH: Glutathione
ROS: Reactive oxygen species
PCD: Programmed cell death
Fer-1: Ferrostatin-1
Lip-1: Liproxstatin-1
ZVF: Z-VAD-FMK
Nec-1: Necrostatin-1
NAC: N-acetylcysteine
OCR: Oxygen consumption rate
AST: Aspartate aminotransferase
ALT: Alanine aminotransferase
IHC: Immunohistochemistry
