# Supplementary material for: SLC25A39 facilitates Sorafenib resistance in hepatocellular carcinoma by inhibiting mitochondrial oxidative stress-induced ferroptosis
Source: Cancer Cell Int. 2026 Jan 6;26:63. doi: 10.1186/s12935-025-04151-9 (PMC12870238; doi:10.1186/s12935-025-04151-9)
Supplement: Supplementary file 1 — Supplementary Material 1 [file 12935_2025_4151_MOESM1_ESM.doc]

**Supplemental information**

**SLC25A39 facilitates cell survival and sorafenib resistance in hepatocellular carcinoma by inhibiting mitochondrial oxidative stress-induced ferroptosis**

**Supplemental figures**

**Figure S1.** **Pan-cancer analysis for the expressions and clinical implications of SLC25A39 using the online Sangerbox 3.0 database. (A)** Immunofluorescence (IF) assay was conducted to test the mitochondrial localization of SLC25A39 in HCC cells (Tom20 serve as a mitochondrial-specific protein) (B) The expressions of SLC25A39 were analyzed using the TCGA database. (C) The clinical implications of SLC25A39 were analyzed using the TCGA database.


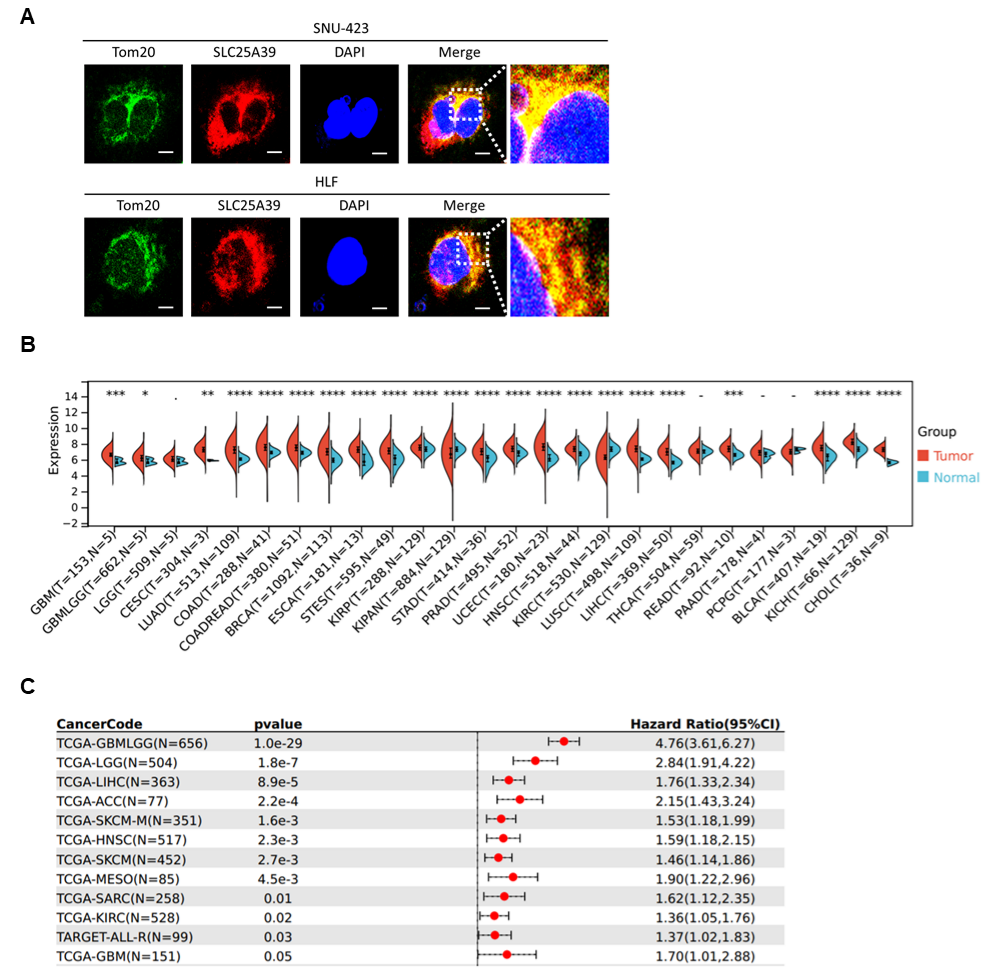


**Figure S2.** Effect of SLC25A39 silencing on the migration (A), invasion (B) and proliferation (C) of SNU-449 and HLF cells were determined by wound healing, matrigel-coated transwell invasion and EdU staining assays.

**
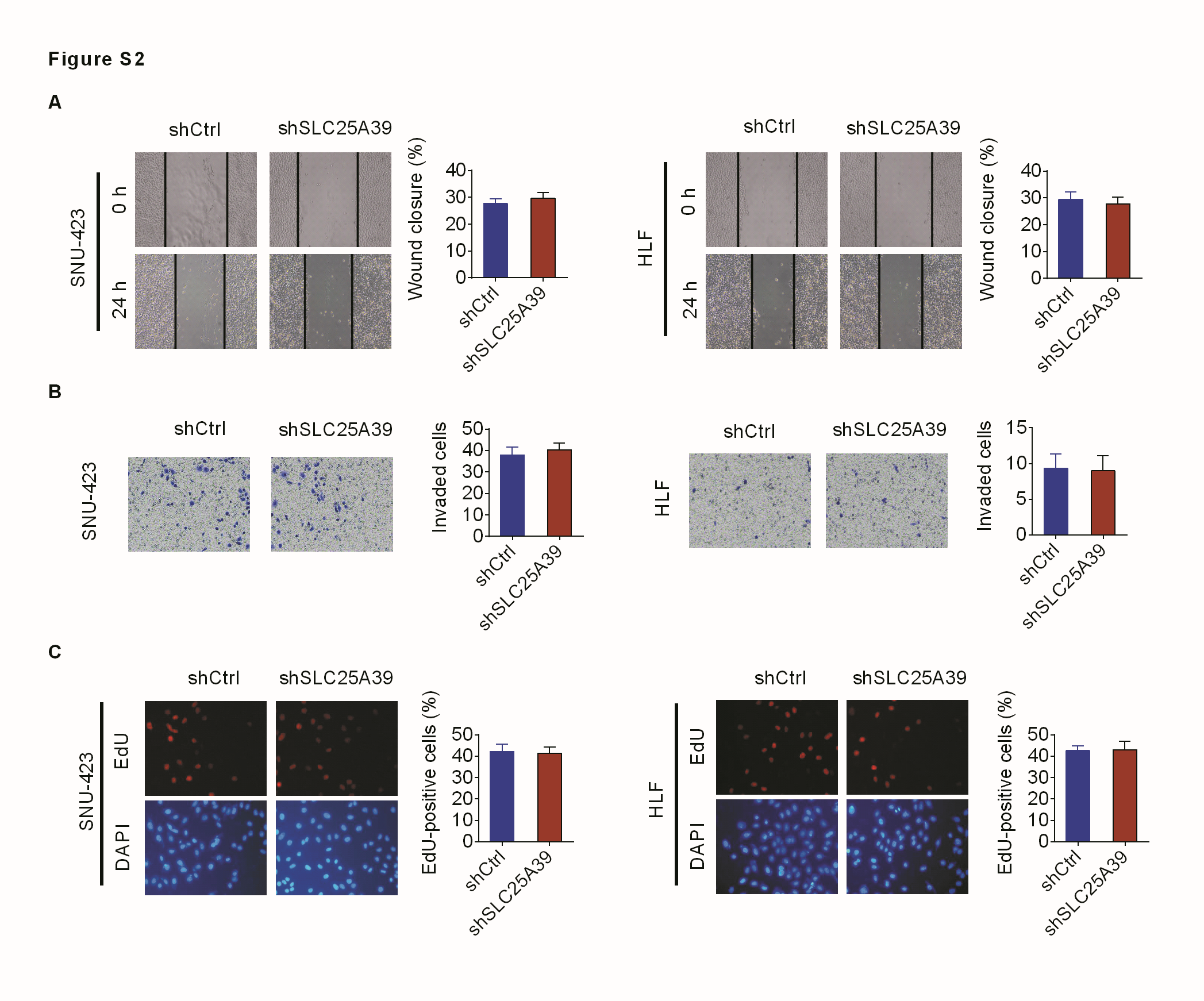
**

**Figure S3. SLC25A39 overexpression promoted the viability and colony formation of THLE-2 cells. (A and B)** SLC25A39 overexpression in THLE-2 cells was verified by qRT-PCR (A) and western blotting (B) assays. **(C and D)** CCK-8 (C) and colony formation (D) assays were conducted in THLE-2 cells upon SLC25A39 overexpression.

**
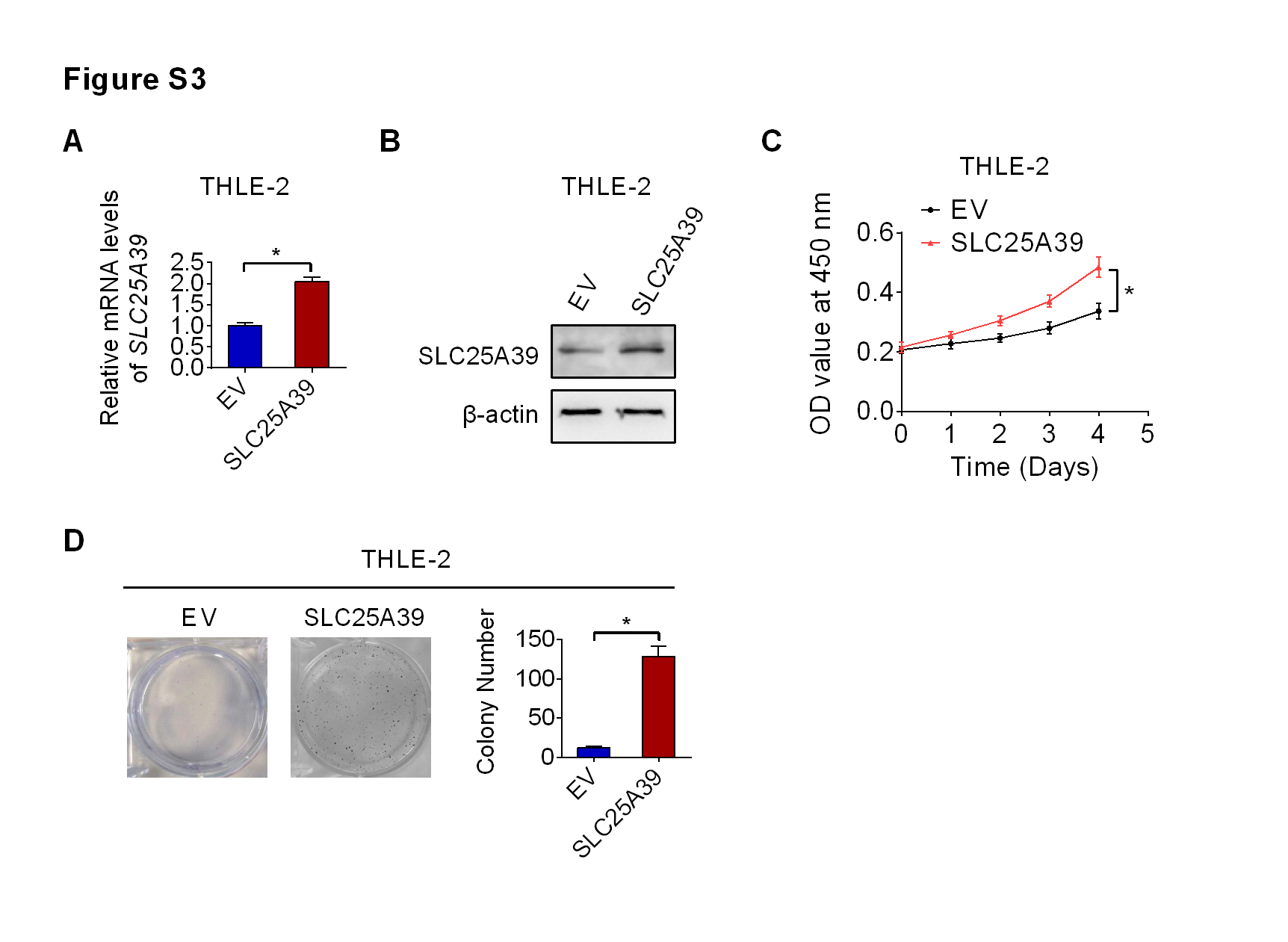
**

**Figure S4. (A-C)** The effects of SLC25A39 silencing or overexpression on mitochondrial oxygen consumption rate (OCR) (A), membrane potential (B, Scale bars=10 µm) and ATP production (C) were evaluated.

**
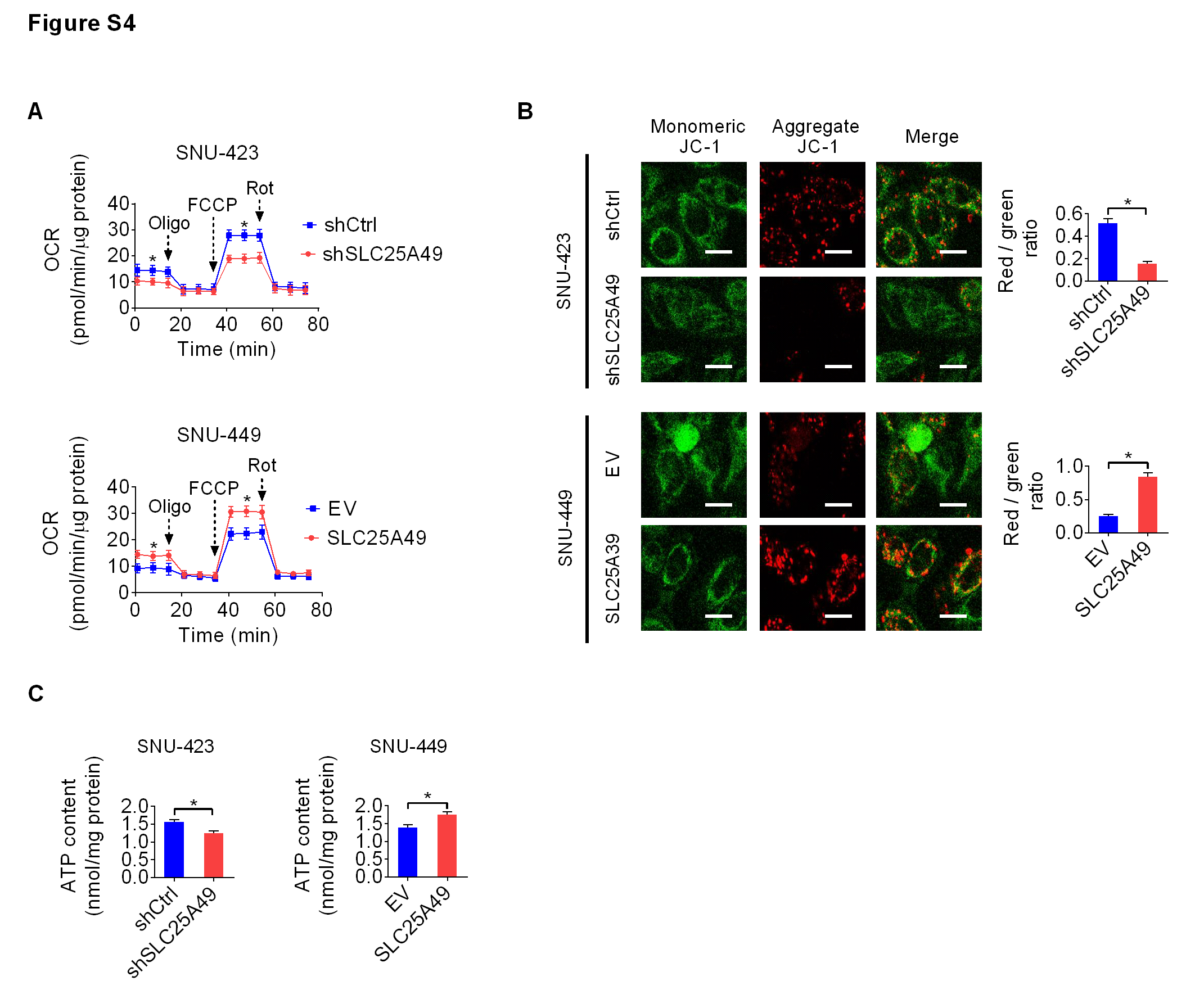
**

**Figure S5.** (A) Successful development of sorafenib-resistant SNU-423 and HLF cells was confirmed by sorafenib IC50 analysis. (B) Flow cytometry analysis of cell death upon treatment of sorafenib (10 µM) for 12 hours in parent and sorafenib-resistant SNU-423 and HLF cells.

**
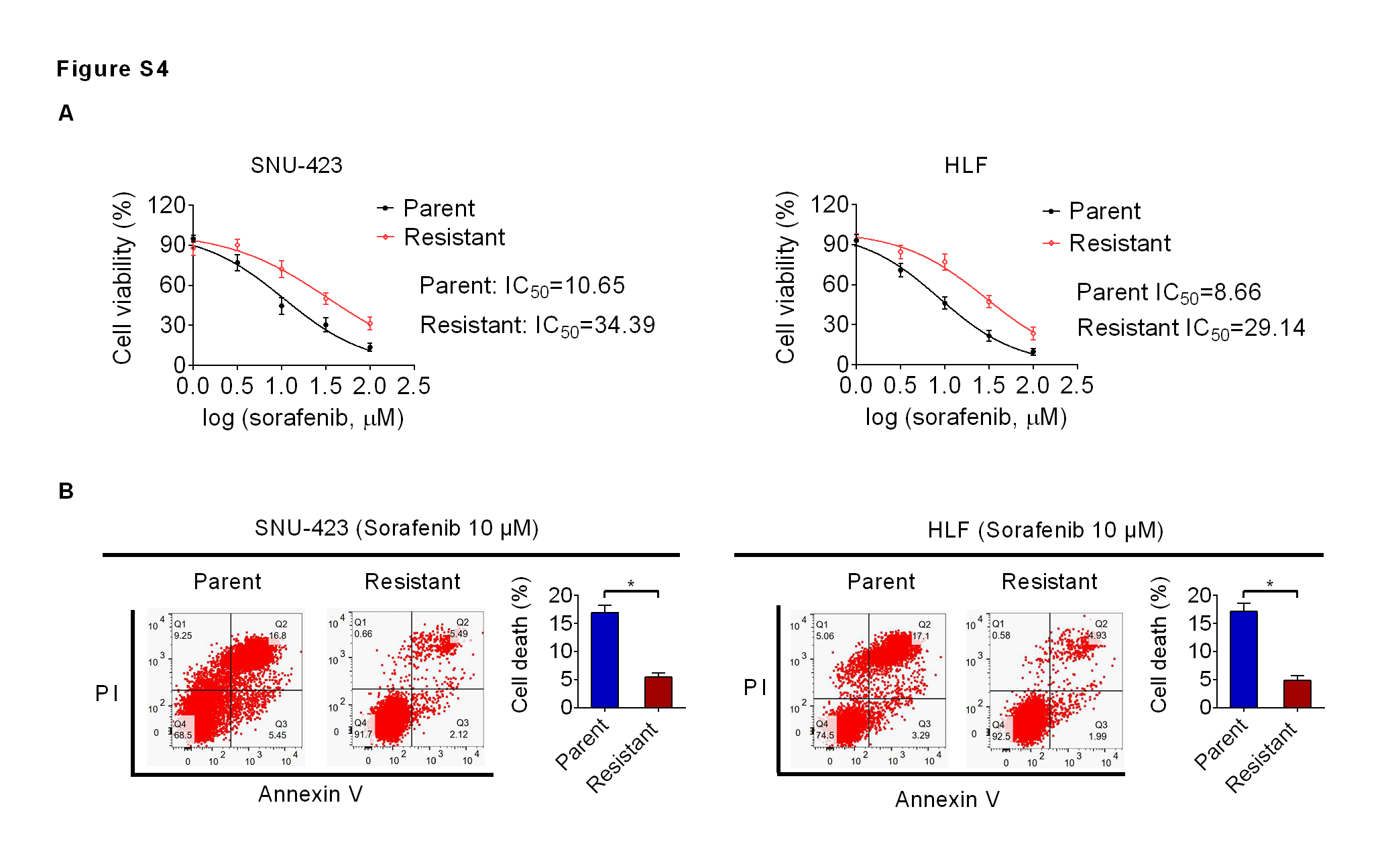
**

**Figure S6.** Serum levels of alanine aminotransferase (ALT) (A) and aspartate aminotransferase (AST) (B) were detected in nude mice with indicated treatment. (C-F) Therapeutic efficacy of targeting SLC25A39 or/and induction of ferroptosis in sorafenib-resistant cell–derived HCC mouse models **(C,** Schematic description of the animal experimental design; Representative photos (D, Scale bars=200 µm), sizes (E) and weights (F) of tumors from the nude mice.

**
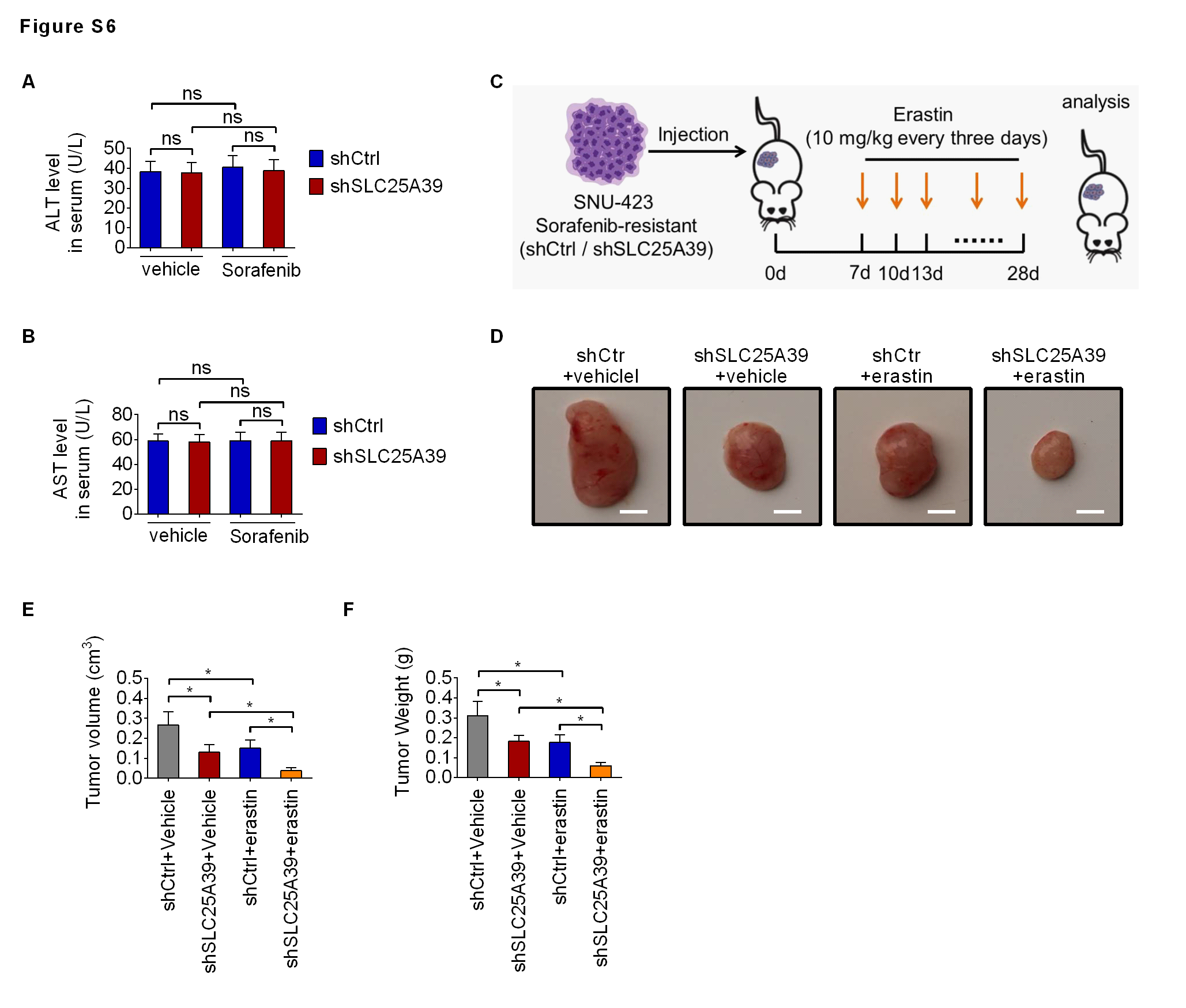
**

**Supplementary Tables**

**Table S1. P**rimers used in qRT-PCR assay.

| **Gene** | **Forward Primer** | **Reverse Primer** |
| --- | --- | --- |
| *SLC25A39* | TCGTGAAGATCGTGAGGCAC | GGCTCGACCACACAGGAAG |
| *β-actin* | AGGCACCAGGGCGTGAT | GCCCACATAGGAATCCTTCTGAC |

**Table S2.** Primary antibodies used in this study.

| **Antibody** | **Company (Cat. No.)** | **Working dilutions** |
| --- | --- | --- |
| SLC25A39 | Novus (NBP2-94802) | IHC, 1/300; WB, 1/1000; IF, 1:200 |
| Ki-67 | Abcam (ab15580) | WB, 1/1000 |
| 4-HNE | Abcam (ab48506) | IHC, 1/200 |
| MDA | Abcam (ab27642) | IHC, 1/300 |
| cleaved caspase-3 | Cell signaling technology (#9661S) | IHC, 1/400 |
| GPX4 | Proteintech (30388-1-AP) | WB, 1/1000 |
| ACSL4 | Proteintech (22401-1-AP) | WB, 1/1000 |
| Tom20 | Proteintech (11802-1-AP) | IF, 1:150 |
| β-actin | Proteintech (20536-1-AP) | WB, 1/1000 |

**Table S3. Correlation between the expressions of SLC25A39 and clinicopathologic features of HCC patients (n=218).**

| **Variables** | **No. of cases (%)** | **SLC25A39 expression** | | ***p* value** |
| --- | --- | --- | --- | --- |
|  |  | **Low** | **High** |  |
| All | 218 (100%) | 109 | 109 |  |
| Age |  |  |  |  |
| <55 | 94 (43.1%) | 50 | 44 | 0.494 |
| >=55 | 124 (56.9%) | 59 | 65 |  |
| Gender |  |  |  |  |
| Female | 37 (17.0%) | 22 | 15 | 0.279 |
| Male | 181 (83.0%) | 87 | 94 |  |
| HBV |  |  |  |  |
| Negative | 26 (11.9%) | 8 | 18 | 0.058 |
| Positive | 192 (88.1%) | 101 | 91 |  |
| alpha-fetoprotein (ug/ml) |  |  |  |  |
| <200 | 125 (57.3%) | 65 | 60 | 0.584 |
| >=200 | 93 (42.7%) | 44 | 49 |  |
| Maximum diameter of lesion |  |  |  |  |
| <5 | 124 (56.9%) | 72 | 52 | ***0.009*** |
| >=5 | 94 (43.1%) | 37 | 57 |  |
| PVTT |  |  |  |  |
| No | 187 (85.8%) | 98 | 89 | 0.120 |
| Yes | 31 (14.2 %) | 11 | 20 |  |
| TNM stage |  |  |  |  |
| I+ II | 171 (78.4 %) | 88 | 83 | 0.510 |
| III+ IV | 47 (21.6%) | 21 | 26 |  |
| Differentiation grade |  |  |  |  |
| I+ II | 73 (33.5%) | 43 | 30 | 0.085 |
| III | 145 (76.5%) | 66 | 79 |  |

**Abbreviations**: TNM, tumor-nodes-metastases; PVTT, portal vein tumor thrombosis
